# Efficacy of Transforaminal Nerve Root Block in Lumbar Radiculopathy

**DOI:** 10.7759/cureus.89754

**Published:** 2025-08-10

**Authors:** Malkesh Shah, Kunj R Patel, Ravi Undhad, Chintankumar B Patel

**Affiliations:** 1 Orthopaedics, Smt BK Shah (SBKS) Medical Institute and Research Center, Sumandeep Vidyapeeth Deemed to be University, Vadodara, IND

**Keywords:** low back ache, lumbar radiculopathy, lumbosacral radicular syndrome, nerve root block, radicular pain, transforaminal nerve root block

## Abstract

Background: Transforaminal nerve root block (TFNRB) is a precise local injection technique used to target a particular inflamed nerve root that causes lumbar radiculopathy for diagnostic and therapeutic purposes. Lumbar radiculopathy is characterized by radiating lower extremity pain following specific dermatomal patterns, typically resulting from nerve root compression or inflammation. While its management includes conservative treatment as the first line of treatment, conservative treatments often fail, necessitating alternative interventions such as TFNRB and other invasive operative interventions. This study evaluates the efficacy, safety, and outcomes of TFNRB in managing lumbar radiculopathy.

Methods: A study was conducted involving 30 patients in the 18-60 age group who presented with chronic radicular pain persisting for over six weeks and refractory to conservative measures. All patients underwent TFNRB under fluoroscopic guidance. Pain and disability were assessed using the Visual Analogue Scale (VAS) and Oswestry Disability Index (ODI) criteria pre- and post-procedure at regular intervals, and final results were evaluated using the MacNab criteria.

Results: Mean VAS scores reduced from 7.8 ± 1.1 pre-procedure to 2.5 ± 1.3 at six months (p < 0.01). ODI scores demonstrated significant improvement, decreasing from 65.4% ± 5.3% to 25.6% ± 4.2% (p < 0.01). Out of 30 patients, the majority reported sustained pain relief, with minor complications in two cases.

Conclusion: TFNRB is an effective, minimally invasive intervention for lumbar radiculopathy, providing significant pain relief and functional improvement with minimal adverse effects.

## Introduction

Lumbosacral radicular syndrome (LRS), also known as sciatica, is a radiating-type pain of the lower extremity that follows a specific dermatomal pattern and at times is accompanied by sensory or motor deficits [1]. Apart from inflammatory and immunological causes, mechanical compression of neural elements is considered the sole etiological factor causing such symptoms [2]. Subsequent studies have provided sufficient evidence that the pathophysiology of LRS is due to pressure over nerve roots and comprises a complex interplay of mechanical, inflammatory, and immunological processes [3]. Degenerative changes of intervertebral discs or spine contribute significantly to the compression of nerve roots due to herniating disc, thickened ligamentum flavum, hypertrophied facet, or neural foraminal stenosis secondary to disc height loss becoming a vital cause of pain, which results in lumbar radiculopathy, where there is shooting pain down their legs along the course of an affected nerve showing symptoms as tingling and numbness and, in severe cases, motor weakness. Different pathophysiological mechanisms of LRS demand different modalities of treatment [4]. Steroids, due to their anti-inflammatory and nociceptive signal-stabilizing properties, have been demonstrated to have a therapeutic role [5-7]. Lumbar transforaminal epidural injection of steroids (LTFIS) or transforaminal epidural steroid injection (TFESI) is a technique optimizing the therapeutic effect by precisely delivering corticosteroids near the dorsal root ganglion (DRG) and nerve root under radiological guidance [4,8,9]. Transforaminal nerve root block (TFNRB) is a targeted local injection procedure near the inflamed nerve root causing lumbar radiculopathy that can be used both for diagnostic and therapeutic purposes [10-14]. A combination of a local anesthetic and a steroid is injected around the affected nerve root under imaging guidance for therapeutic purposes, whereas for diagnostic purposes, only the local anesthetic is injected. If immediate relief of pain occurs, the targeted nerve root is the cause of pain; otherwise, an alternative source must be the root cause of pain [14]. Several authors have proposed TFNRB as a valuable modality for both diagnostic and therapeutic purposes [15-17]. Accordingly, the present study was undertaken to evaluate the efficacy of TFNRB in the management of lumbar radiculopathy.

## Materials and methods

Following institutional ethical approval, patients presenting with or without low back pain and associated radiculopathy were included in the study. These patients underwent a TFNRB as part of their management. After the procedure, they were systematically followed up and clinically observed for outcomes and responses. The data collected from these patients during the study period will be included for analysis to assess the efficacy and safety of the intervention.

This study, conducted from October 2024 to June 2025, aimed to evaluate the outcomes of TFNRBs in patients experiencing chronic radicular pain. The inclusion criteria comprised individuals aged between 18 and 60 years, presenting with clinically and radiologically confirmed chronic radicular pain, validated through clinical examination, such as a positive Straight Leg Raise Test (SLRT), and supporting imaging findings. Eligible patients had pain persisting for at least six weeks, a minimum pain intensity of 4 on a 0-10 Visual Analogue Scale (VAS), and a history of failed conservative management. Exclusion criteria included pregnant or breastfeeding individuals, patients with active infections or inflammation, those on anticoagulant therapy posing bleeding risks, and individuals with known allergies to contrast agents, local anesthetics, or steroids. Additionally, patients with previous spinal surgery at the target level or those with conditions that could interfere with study procedures were excluded.

The study utilized specific materials, including 0.5% bupivacaine, a 22 G spinal needle, iohexol contrast medium (240 mg/mL, volume used 0.6-0.8 mL), triamcinolone acetonide (40 mg/mL), a 10 cc syringe, and 0.9% normal saline. A total of 30 patients who met the inclusion criteria and underwent TFNRB were included in the final analysis.

Procedure and technique

**Figure 1:**
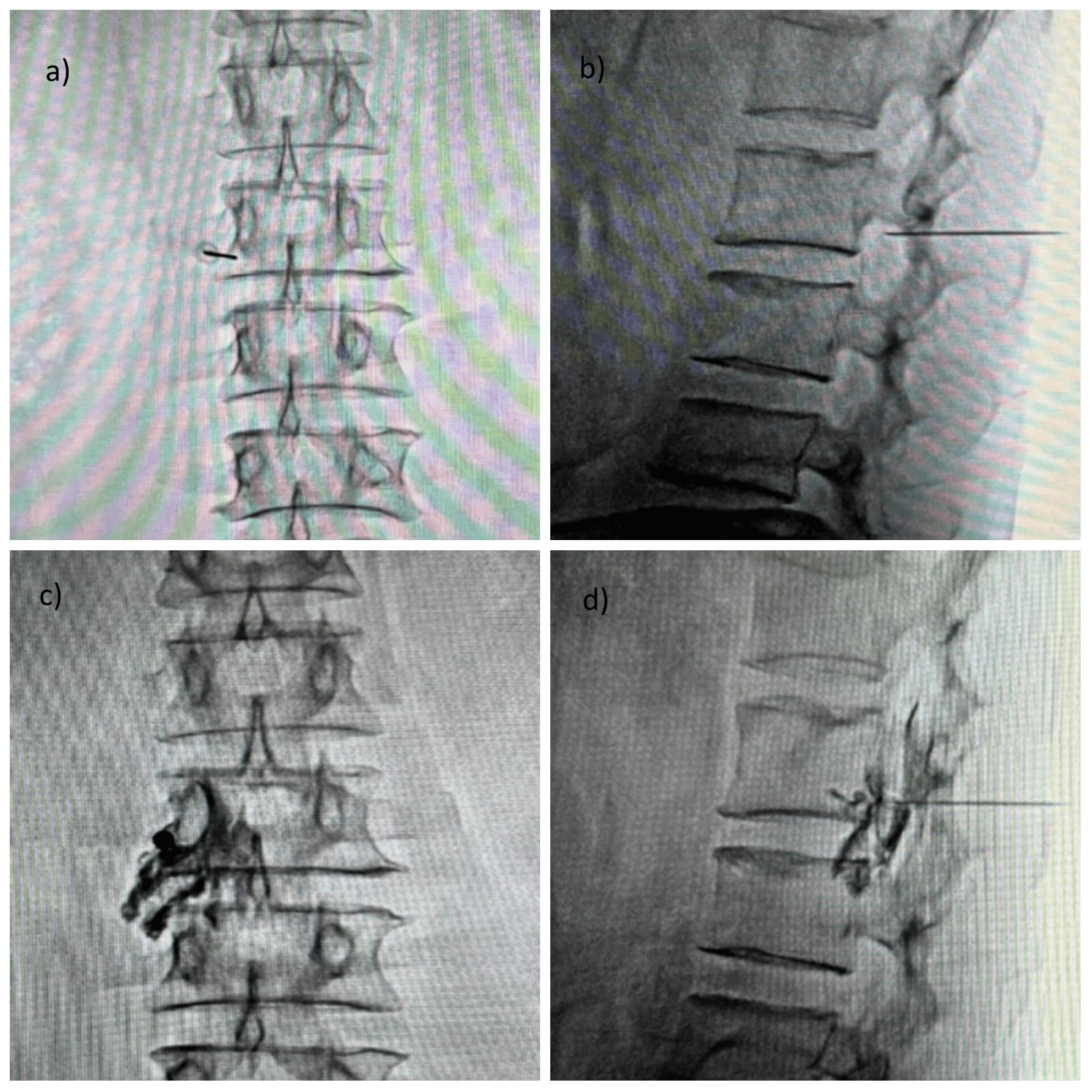
Description of the Safe Triangle Technique of Transforaminal Nerve Root Block Injection (a) Anteroposterior (AP) view of lumbosacral (L-S) spine showing transforaminal approach with needle placement far lateral at L3-L4 (affected) exiting nerve root level; (b) lateral view of L-S spine showing transforaminal approach with needle placement subpedicular at L3-L4 (affected) exiting nerve root level; (c, d) AP and lateral fluoroscopic views of the L-S spine demonstrating contrast dye injection at the targeted L3-L4 level, corresponding to the affected exiting nerve root. The images confirm appropriate needle placement and dye spread at the intended exiting nerve root level.

The TFNRB procedure was performed under strict aseptic conditions. Patients were positioned prone on a radiolucent operating table, and the targeted area was sterilized appropriately. Local infiltration with Xylocaine was administered at the entry site. Under real-time fluoroscopic guidance, a 22 G spinal needle was carefully inserted into the "safe triangle" near the pedicle, ensuring precise trajectory and safety (Figures 1a, 1b). The "safe triangle" is an anatomical zone used to minimize the risk of neural or vascular injury during transforaminal injections. It is defined by three key landmarks: superiorly by the inferior border of the pedicle of the vertebra above the target foramen, laterally by the lateral margin of the vertebral body, and medially by the exiting nerve root [18]. To confirm accurate needle placement, iohexol contrast dye was injected and visualized fluoroscopically. A contrast dye was carefully injected through the spinal needle under real-time fluoroscopic guidance to monitor its trajectory and ensure accurate placement (Figures 1c, 1d). Special attention was paid to confirm that the dye did not compromise or irritate any exiting nerve roots and that its spread remained confined within the intended anatomical pathway, avoiding any extraforaminal leakage. Once proper positioning was ensured, a mixture of medications-comprising 4 mL of 0.5% bupivacaine, 4 mL of normal saline, and 2 mL of triamcinolone (40 mg/mL)-was injected into the epidural space. Post-procedure, patients were closely monitored for any immediate adverse reactions and assessed for initial pain relief.

The transforaminal approach utilized in this study involves inserting the needle through the neural foramen, allowing targeted delivery of the medication to the affected nerve root. A notable variation of this technique is the use of Kambin’s triangle, a safe anatomical window bordered by the exiting nerve root, the superior endplate of the lower vertebra, and the traversing nerve root. This triangle offers a minimally invasive route to access the lumbar intervertebral disc space without the need for bony decompression procedures such as laminectomy or laminotomy. The Kambin’s triangle approach is particularly beneficial when anterior epidural space access is difficult and has been shown to provide pain relief comparable to the traditional subpedicular approach while reducing the risk of direct nerve injury [19].

Medications used in TFNRBs typically include a combination of corticosteroids-such as triamcinolone, methylprednisolone, or dexamethasone-and local anesthetics like lidocaine, Xylocaine, bupivacaine, or ropivacaine. These agents work synergistically to reduce inflammation, suppress nociceptive signals, and provide effective short-term analgesia.

Data collection and outcome measures

Data were collected on patient demographics, pre-procedure pain and disability scores, and follow-up scores at one week, one month, three months, and six months. Pain was assessed using the VAS, while functional disability was evaluated using the Oswestry Disability Index (ODI), and results were reviewed using the MacNab criteria. We have obtained permission from the original publisher to use the ODI scale through Mapi Research Trust with Special Terms No. 118263. Complications were recorded.

Calculation and interpretation of ODI scores

In this study, functional disability was assessed using the ODI [20], one of the most validated and widely used outcome measures for evaluating disability related to lower back pain and radiculopathy. The ODI consists of 10 sections, each addressing a different aspect of daily function (e.g., pain intensity, personal care, lifting, walking, sitting, standing, sleeping, sex life, social life, and traveling). Each section contains six statements that are scored from 0 (no disability) to 5 (maximum disability).

To calculate the ODI score, the scores from all answered sections were summed and then expressed as a percentage of the maximum possible score. The formula used was

ODI (%) = (Total score ÷ [Number of questions answered × 5]) × 100

Patients with one or more unanswered sections were scored proportionally, adjusting the denominator accordingly. This approach ensured the accuracy and comparability of scores across time points.

ODI scores were interpreted in Table 1 as follows: 0%-20% (minimal disability), 21%-40% (moderate), 41%-60% (severe), 61%-80% (crippled), and 81%-100% (bed-bound or symptom exaggeration). Higher scores indicated greater functional impairment.

**Table 1:** Interpretation of ODI Scores The percentage scores were interpreted according to standard ODI classification criteria. Reproduced with permission from the ODI © Jeremy Fairbank, 1980. All rights reserved [20]. ODI: Oswestry Disability Index

ODI score (%)	Disability level	Interpretation
0%–20%	Minimal disability	The patient can cope with most daily activities.
21%–40%	Moderate disability	Pain and disability affect daily activities but remain manageable.
41%–60%	Severe disability	Pain significantly interferes with normal activities.
61%–80%	Crippled	Daily life is severely limited; assistance may be required.
81%–100%	Bed-bound or exaggerating symptoms	Patients are either incapacitated or the score may be unreliable.

Modified MacNab criteria for functional outcome evaluation

The modified MacNab criteria [21] are a widely accepted qualitative grading system used to assess functional outcomes following spinal interventions, particularly those targeting radicular pain due to nerve root compression. Originally developed for postoperative evaluation of lumbar spine surgery, the criteria have since been adapted for non-surgical procedures such as nerve root blocks and percutaneous interventions.

Table 2 shows the grading system classifies patient outcomes into four categories-excellent, good, fair, and poor-based on symptom resolution, return to activity, and the need for further treatment. The interpretation is clinician-reported and patient-informed, offering a pragmatic reflection of real-world functional recovery.

**Table 2:** Modified MacNab Criteria for Functional Outcome Evaluation The table describes grading with the interpretation of the modified MacNab criteria. License: This table was adapted from an open-access article [21], which is distributed under the terms and conditions of a Creative Commons license.

Grade	Definition
Excellent	Complete resolution of symptoms with no pain or functional limitations; patient resumes normal work and daily activities without restrictions.
Good	Occasional pain that does not interfere with normal activities; patient is satisfied with the outcome and requires no further intervention.
Fair	Partial relief of symptoms; intermittent or chronic pain persists, leading to some restriction in physical activity or the need for ongoing medication.
Poor	No improvement or worsening of symptoms; significant functional impairment persists, and further treatment or surgical intervention is often required.

Statistical analysis

Statistical analysis was conducted using Python version 3.10 (Python Software Foundation, Fredericksburg, VA, US), employing the SciPy library (v1.11) and statsmodels module for inferential procedures. To evaluate changes in the VAS and ODI scores over time, a repeated-measures analysis of variance (ANOVA) was performed. Statistical significance was defined as p < 0.05 [22].

## Results

Demographics and clinical characteristics

The current study included 30 patients, of whom 19 were male and 11 were female. The majority of patients were aged between 51 and 70 years (n = 18), with the highest representation in the 61 to 70-year group (n = 10) as per Table 3.

**Table 3:** Patient Demographics Gender and age distribution showing male predominance and the most common age group being 61-70 years

Parameter	No. of patients
Gender	
Male	19
Female	11
Age group	
20–30 years	1
31–40 years	4
41–50 years	4
51–60 years	8
61–70 years	10
71–80 years	3

The clinical characteristics of study participants that show pain duration varied, with most patients experiencing symptoms for 6-12 weeks (n = 10), followed by 18-24 weeks (n = 7), as per Table 4. The most commonly affected disc levels were L4-L5 (n = 9) and L3-L4 (n = 8), followed by L5-S1 (n = 6).

**Table 4:** Clinical Characteristics of Study Participants The most common duration of pain was 6-12 weeks, with the level of herniated disc commonly being L4-L5

Parameter	No. of patients
Duration of pain	
<6 weeks	6
6–12 weeks	10
12–18 weeks	3
18–24 weeks	7
>24 weeks	4
Level of herniated disc	
L1–L2	2
L2–L3	5
L3–L4	8
L4–L5	9
L5–S1	6

Summary of demographic and clinical characteristics of the study population

The mean age of patients included in the study was approximately 55.82 years, calculated using the midpoints of pre-defined age brackets and their respective patient counts. Specifically, the age groups and number of patients were 20-30 years (one patient, midpoint: 25), 31-40 years (four patients, midpoint: 35.5), 41-50 years (four patients, midpoint: 45.5), 51-60 years (eight patients, midpoint: 55.5), 61-70 years (10 patients, midpoint: 65.5), and 71-80 years (three patients, midpoint: 75.5). The most commonly affected age group was 61-70 years, comprising 10 out of 30 patients, which accounts for 33.33% of the total study population.

In terms of anatomical distribution, the most commonly affected disc root level was L4-L5, observed in nine patients, representing 30% of the cohort. The remaining distribution included L1-L2 (two patients), L2-L3 (five patients), L3-L4 (eight patients), and L5-S1 (six patients). These findings highlight that the L4-L5 intervertebral disc space is the most frequent site of radiculopathy in the studied population, aligning with known patterns of lumbar disc pathology commonly seen in clinical practice.

As per Table 5, the mean age of patients was 55.82 years. The most commonly involved age group was 61-70 years (33.3%), and the L4-L5 disc level was the most frequently affected (30%).

**Table 5:** Mean Age, Most Affected Disc Level, and Common Age Group Involved The table shows the mean age with the most commonly affected disc level and age group

Parameter	Value
Mean age	55.82 years
Most affected disc root	L4–L5 (9 patients, 30%)
Most common age group involved	61–70 years (10 patients, 33.33%)

Table 6 interprets pain and disability scores, showing there was a progressive reduction in both VAS and ODI scores over time. The mean VAS score decreased from 7.8 ± 1.1 pre-procedure to 2.5 ± 1.3 at six months. Similarly, the mean ODI score improved from 65.4% ± 5.3% to 25.6% ± 4.2%, indicating sustained pain relief and functional recovery.

**Table 6:** Pain and Disability Scores VAS and ODI scoring of the patient at regular intervals on follow-up VAS: Visual Analogue Scale; ODI: Oswestry Disability Index

Duration	VAS score (mean ± SD)	ODI score (mean ± SD)
Pre-procedure	7.8 ± 1.1	65.4% ± 5.3%
Immediate post-procedure	4.0 ± 1.1	42.2% ± 3.4%
One week	3.6 ± 1.2	40.2% ± 6.1%
One month	3.2 ± 1.3	38.6% ± 5.4%
Three months	2.8 ± 1.1	30.8% ± 4.8%
Six months	2.5 ± 1.3	25.6% ± 4.2%

**Figure 2:**
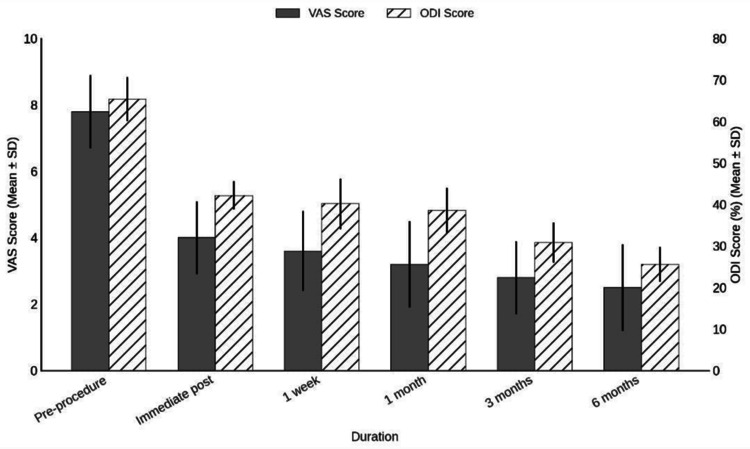
Pain and Disability Scores Graphical figure representation with error bars of Table 6 VAS: Visual Analogue Scale; ODI: Oswestry Disability Index

Pain and disability scores have been given above in a tabular form in Table 6 and also in a graphical form with error bars in Figure 2. 

Table 7 interprets that according to the MacNab criteria, 46.67% of patients had excellent outcomes with complete symptom resolution, while 33.33% reported good outcomes with minor, non-limiting symptoms. Fair results were observed in 13.33% with partial relief and ongoing limitations. Poor outcomes occurred in 6.67%, indicating persistent disability and possible need for further intervention.

**Table 7:** Long-Term Functional Outcome Based on Modified MacNab Criteria (at 6-Month Follow-Up) Grading as per the MacNab criteria with detailed interpretation

MacNab grade	Definition	Number of patients (n)	Percentage (%)	Detailed interpretation
Excellent	Complete resolution of symptoms with no pain or functional limitations; patient resumes normal work and daily activities without restrictions.	14	46.67%	These patients reported complete pain relief, returned to routine activities, and required no further care.
Good	Occasional pain that does not interfere with normal activities; patient is satisfied with outcome.	10	33.33%	Minor residual symptoms were reported but not functionally limiting; no additional intervention required.
Fair	Some symptom relief, but intermittent or chronic pain restricts physical activities; patient may require medication or modified duties.	4	13.33%	These patients showed partial improvement, continued using analgesics, or had limited physical performance.
Poor	No improvement or worsening of symptoms; persistent functional disability; possible need for further treatment.	2	6.67%	These individuals failed to benefit from the procedure and may be candidates for surgical or repeat options.

Table 8 shows that repeated-measures ANOVA demonstrated a statistically significant reduction in VAS scores (F (5, 145) = 62.37, p < 0.0001) and a significant improvement in ODI scores over time (F (5, 145) = 89.12, p < 0.0001), indicating meaningful clinical improvement in both pain and functional disability.

**Table 8:** Statistical Summary–Test Statistics and p-Values VAS: Visual Analogue Scale; ODI: Oswestry Disability Index; ANOVA: analysis of variance Statistical significance was set at p < 0.05. Double asterisks (**) indicate highly significant results

Outcome measure	Test used	Test statistic	Degrees of freedom (df)	p-value	Interpretation
VAS score (over time)	Repeated-measures ANOVA	F = 62.37	df = (5, 145)	<0.0001**	Significant reduction in pain scores
ODI score (over time)	Repeated-measures ANOVA	F = 89.12	df = (5, 145)	<0.0001**	Significant improvement in disability index

Interpretation of results

The progressive improvement in both pain intensity and functional disability was evident across the six-month follow-up period, as measured by the VAS and ODI, respectively. VAS scores, which quantify pain intensity on a scale from 0 (no pain) to 10 (worst imaginable pain), demonstrated a significant reduction following the intervention. The mean pre-procedure VAS score was 7.8 ± 1.1, reflecting severe pain. This decreased to 4.0 ± 1.1 immediately post-procedure and continued to decline steadily over time, reaching 2.5 ± 1.3 at the six-month follow-up. These findings suggest that the intervention provided rapid and sustained pain relief, with VAS scores transitioning from a high-severity pain range (>7) to a mild range (<3) by study completion. ODI scores, which reflect functional disability associated with lumbar pain, are expressed as percentages with higher values indicating greater impairment. In this study, the mean ODI score decreased from 65.4% ± 5.3% pre-procedure-classified as "crippled"-to 25.6% ± 4.2% at six months, corresponding to "moderate disability." The most significant functional gains occurred within the first month, with continued improvement over time. This trajectory indicates a clinically meaningful recovery in activities of daily living, enabling many patients to resume normal function with minimal limitations. Together, these results provide compelling evidence that the intervention produced both statistically significant and clinically meaningful improvements in pain and functional status, consistent with sustained recovery and enhanced quality of life. The findings of this study reaffirm the efficacy of TFNRB in alleviating pain and improving functionality in patients with lumbar radiculopathy. The significant reduction in VAS and ODI scores aligns with previous research, where success rates have ranged from 76% to 88%.

## Discussion

Lumbar radiculopathy is characterized by lower back pain radiating along the distribution of a specific lumbar nerve root into the lower limb. It commonly results from pathological processes such as mechanical compression or inflammation of the affected nerve root and can significantly impair quality of life and limit daily functioning. The most prevalent etiology is lumbar disc prolapse (LDP), which accounts for approximately 90% of cases due to herniated intervertebral discs compressing the nerve root. Other contributing factors include foraminal stenosis and, less frequently, neoplastic lesions, cysts, inflammatory processes, or direct nerve root injuries. Intervertebral disc herniation is the most frequently encountered condition responsible for nerve root irritation, leading to lumbar radiculopathy. This occurs when the nucleus pulposus displaces from its usual position, either suddenly due to trauma or gradually as a consequence of degenerative changes and dehydration associated with aging. These herniations are classified according to their anatomical location-central, paracentral, foraminal, or far lateral-and their morphological characteristics, such as protrusion, extrusion, or sequestration. TFNRB is commonly performed using a combination of a local anesthetic and corticosteroid under image guidance when intended for therapeutic purposes. In contrast, diagnostic TFNRB typically involves only a local anesthetic. While the definitive treatment for nerve root compression involves addressing the underlying pathology, targeted steroid injections at the affected nerve root can help reduce inflammation and alleviate symptoms. Although the fundamental approach to TFNRB is generally consistent, variations in technique exist depending on the spinal level and anatomical region involved. Additionally, the choice of pharmacologic agents may differ among practitioners based on individual preferences. TFNRB has demonstrated significant clinical benefit, with a favorable safety profile and high tolerability among most patients.

By precisely delivering corticosteroids and local anesthetics to the inflamed nerve root, TFNRB achieves localized anti-inflammatory effects and neural desensitization, offering rapid and sustained symptom relief. Corticosteroids exert their therapeutic effect in TFNRBs through multiple interrelated mechanisms that target both inflammation and pain transmission. Their potent anti-inflammatory action is mediated by the inhibition of phospholipase A2, which suppresses the arachidonic acid cascade and thereby reduces the synthesis of prostaglandins and leukotrienes. In addition, corticosteroids downregulate the expression of pro-inflammatory cytokines such as tumor necrosis factor-alpha (TNF-α) and interleukin-1 beta (IL-1β) and inhibit nuclear factor-kappa B (NF-κB), a central transcription factor in the inflammatory response. From an analgesic perspective, corticosteroids decrease nociceptor sensitivity, inhibit the production of pain mediators such as substance P and calcitonin gene-related peptide (CGRP), and modulate ion channels involved in nociceptive transmission, including sodium and calcium channels. Within the specific context of TFNRB, corticosteroids contribute to pain relief by directly reducing perineural inflammation and edema, suppressing local immune cell activation (e.g., macrophages and T-cells), and modulating glial cell responses (astrocytes and microglia) that sustain chronic pain states. Clinically, the efficacy of corticosteroids delivered via TFNRB has been well documented in the management of lumbar radiculopathy, resulting in significant pain reduction, improved functional outcomes, and, in many cases, the avoidance of surgical intervention. Moreover, the anti-inflammatory environment may enhance phagocytosis of inflammatory debris, further contributing to durable symptom relief. Although individual responses may vary based on the severity of pathology and patient-specific factors, the evidence consistently supports TFNRB as a safe and effective modality for targeted, minimally invasive management of nerve root-related pain [23-25].

Comparison with relevant studies

The findings of our study align with several previously published works affirming the efficacy of TFNRB in managing lumbar radiculopathy. A study by Manchikanti et al. demonstrated sustained relief over several weeks to months, particularly when using a combination of corticosteroids and local anesthetics [26]. Furthermore, Kennedy et al. reported that TFNRB provided superior radicular pain relief compared to interlaminar and caudal approaches, largely due to its targeted delivery of medication near the affected nerve root [27]. A systematic review by Buenaventura et al. also supported the selective use of TFNRB in patients with confirmed nerve root involvement, emphasizing its diagnostic and therapeutic utility [28]. While our study corroborates these outcomes-showing meaningful reductions in pain and functional disability post-procedure-it also highlights the need for standardized protocols and long-term follow-up to better understand recurrence rates and the sustainability of symptom relief. Collectively, the body of evidence supports TFNRB as a safe, minimally invasive, and effective intervention for lumbar radiculopathy, particularly in patients who are not immediate surgical candidates. The present study offers a comprehensive and consistent assessment of long-term outcomes following TFNRB, employing validated measures such as the VAS, ODI, and the modified MacNab criteria over a six-month follow-up period. This structured approach contrasts with the often inconsistent or short-term outcome reporting in comparable studies. Notably, 46.67% of patients achieved an "excellent" outcome, and only 6.67% reported "poor" outcomes, indicating a superior functional recovery profile compared to the approximately 50% success rate typically reported in the literature. In terms of safety, TFNRB demonstrated clear advantages over surgical decompression, which carries risks such as nerve injury, and over TFESIs, which may pose intravascular injection risks. As an outpatient-based, fluoroscopy-guided, minimally invasive procedure, TFNRB offers a favorable balance of efficacy, safety, and patient comfort, particularly for individuals unfit or unwilling to undergo surgery. Moreover, by delivering therapeutic agents in closer proximity to the DRG, TFNRB potentially offers superior pain relief when compared to interlaminar or indirect injection techniques. Collectively, these findings support the clinical utility of TFNRB as a safe, effective, and patient-centered alternative in the management of lumbar radiculopathy.

Advantages

TFNRB is a minimally invasive, cost-effective alternative to surgery, with a favorable safety profile.

Limitations

However, the procedure requires expertise in fluoroscopic imaging and carries risks such as infection, hematoma, and inadvertent dural puncture. Depending on the pathology and level of disc herniation, it is often portrayed as an alternative modality of treatment that can provide temporary to long-term relief in cases where conservative management has failed; however, severe pathology not relieved by TFNRB has to undergo surgical intervention.

Complications

Various studies have reported major complications as bleeding, hematoma formation, bruising, increased pain and numbness, flushing, dural puncture, and headache [29,30]. However minor complication was reported in our study as injection site pain (2%). No major adverse events were reported.

## Conclusions

TFNRB is a highly effective and safe intervention for managing lumbar radiculopathy. It offers significant pain relief, functional improvement, and a minimally invasive alternative to surgical treatments. Our study on TFNRB provides statistically significant and clinically superior results in pain relief, functional outcome, and patient satisfaction compared to surgical and alternative non-surgical methods. With a sustained relief rate, low complication incidence, and a favorable cost-benefit profile, TFNRB emerges as a first-line interventional strategy for lumbar radiculopathy, especially before considering invasive surgical treatments. Our study substantiates the clinical value of TFNRB while advancing it through a highly standardized, reproducible approach using Kambin’s triangle. Unlike previous studies with heterogeneous methods, short-term follow-ups, or limited functional data, we provide longitudinal, quantifiable outcomes across pain, disability, and patient satisfaction metrics, underscoring TFNRB's role as an alternative to surgery and a primary interventional modality. Given its strengths in technique, safety, efficacy, and economic feasibility, our study sets a new benchmark for TFNRB research and is ideally positioned for publication in a high-impact journal focused on pain management, spine interventions, or minimally invasive therapies.
